# Role of the Osteoblast Lineage in the Bone Marrow Hematopoietic Niches

**DOI:** 10.1359/jbmr.090225

**Published:** 2009-02-16

**Authors:** Joy Y Wu, David T Scadden, Henry M Kronenberg

**Affiliations:** 1 Endocrine UnitBoston, Massachusetts, USA; 2 Center for Regenerative Medicine, Massachusetts General HospitalBoston, Massachusetts, USA; 3 Harvard Stem Cell InstituteCambridge, Massachusetts, USA

## INTRODUCTION

In mammals, hematopoiesis shifts to the bone marrow in late embryogenesis, coincident with the appearance of a marrow cavity.(1) There hematopoiesis is sustained throughout adult life unless forced out of the bone marrow into extramedullary sites by pathological conditions. All hematopoietic lineages arise from the hematopoietic stem cell (HSC), and maintenance of HSC self-renewal and differentiation are critically dependent on the presence of a supportive microenvironment, or niche. The existence of such a niche within the bone marrow was first postulated by Schofield,(2) and the vital role of the bone marrow microenvironment has been convincingly shown in the three decades since.

The marrow microenvironment is comprised of cells of multiple lineages, including fibroblast-like cells, adipocytes, osteoblasts, and endothelial cells. Recent studies have begun to highlight the contributions of individual lineages to the hematopoietic niche, with the greatest weight of evidence thus far in support of important roles for osteoblasts and the vasculature.(3–5) In humans, CD146^+^ subendothelial cells have been reported to serve as skeletal progenitors capable of generating cells that organize a hematopoietic microenvironment on transplantation.(6) Subsequent studies have shown that the endosteal surface is rich in vasculature with close approximation of osteoblasts and vessel walls.(7,8) In trabecular bone, it is unlikely that there are physically distinct endosteal and perivascular/vascular niches, although the functional roles of osteoblasts and vascular cells may well differ. This review will focus specifically on the role of cells of the osteoblast lineage within the bone marrow niche. In particular, we will examine the contribution of osteoblasts in supporting hematopoietic stem cells and contrast this with how distinct stages of osteoblast precursors support developing B lymphocytes, one of the best-characterized specific hematopoietic lineages.

## OSTEOBLASTS ARE A DIVERSE POPULATION

To date in the HSC microenvironment literature, osteoblasts have largely been considered as a uniform entity. However, within the osteoblast lineage, there are multiple stages, just as there are intermediates between HSCs and mature B lymphocytes. The osteoblast lineage is derived from a putative mesenchymal stem cell, whose location and identity in vivo has yet to be firmly established. Once committed to the osteoblast lineage, through a process that requires the expression of Runx2, cells differentiate and express progressively more mature markers of osteoblastogenesis including osterix, alkaline phosphatase, and collagen type I.(9) Mature osteoblasts are identified by their cuboidal morphology and localization to the bone surface (endosteal or periosteal), where they secrete extracellular matrix and express markers of terminal differentiation such as osteocalcin. Terminally differentiated osteoblasts themselves have one of three known fates-to become quiescent lining cells, to differentiate further into osteocytes surrounded by mineral matrix, or to die by apoptosis.(10) Whereas osteocytes and mature osteoblasts are easily identifiable in histological sections, little is known about the location and fate of osteoprogenitors in vivo. Osteoblast precursors are likely located in the marrow space, because bone marrow flushed from the diaphysis contains cells capable of adhering to plastic, and, when cultured under osteogenic conditions, a fraction of this adherent population is capable of differentiating into cells that express markers of osteoblasts and mineralize the surrounding matrix.(9) In certain disease states such as severe hyperparathyroidism(11) or fibrous dysplasia,(12) the hematopoietic marrow is replaced by fibroblastoid stromal cells that express markers of the osteoblast lineage.(13,14) Detailed characterization of osteoprogenitors thus far has been limited by a lack of cell surface markers that would allow prospective identification and isolation of defined precursor populations. However, more recently, the development of fluorescent reporter mice in which variants of green fluorescent protein (GFP) are driven by osteoblast stage-specific promoters has revolutionized our ability to study osteoblast differentiation in vivo.(15,16) That each stage of osteoblast differentiation is functionally distinct is underscored by the finding, for example, that, whereas deletion of β-catenin in osteoprogenitors inhibits osteoblast differentiation in favor of a chondrocytic fate,(17–19) deletion in differentiated osteoblasts results in severe osteopenia largely because of enhanced bone resorption.(20,21)

## OSTEOBLASTS SUPPORT HEMATOPOIESIS

A role for osteoblasts in supporting hematopoiesis was first suggested by anatomic evidence. Several groups reported that primitive hematopoietic cells seem to be enriched near the endosteal surface,(22,23) whereas more differentiated progenitors are localized more centrally within the bone marrow space.(24,25) More recently, visualization of labeled hematopoietic stem/progenitor cells (HSPCs) by intravital microscopy showed that HSPCs localized adjacent to endosteal osteoblasts in the settings of HSPC engraftment or expansion; more mature progenitors were positioned further from osteoblasts.(7,8) Moreover, the migration of hematopoiesis from the fetal liver to the bone marrow during embryonic development is critically dependent on normal bone formation and turnover. Mice deficient in Runx2 fail to develop a mineralized skeleton,(26,27) and, in these mice, hematopoiesis shifts to extramedullary organs.(28) In contrast, mice lacking macrophage colony-stimulating factor (M-CSF) have defective osteoclasts and therefore osteopetrosis and also develop extramedullary hematopoiesis.(29)

In vitro, hematopoietic cell differentiation requires the supporting presence of stromal cells,(30) a task that can be performed by cells of the osteoblast lineage.(31,32) A functional role for osteoblasts in regulating hematopoiesis in vivo was shown by targeted ablation of osteoblasts, through gancyclovir treatment of mice expressing herpes simplex thymidine kinase only in differentiated osteoblasts.(33) Loss of osteoblasts led to a dramatic reduction of bone marrow cellularity and resultant extramedullary hematopoiesis, consistent with the loss of the ability of the bone to support hematopoiesis.(34) Ablation of osteoblasts was followed acutely by loss of B-cell lymphopoiesis and erythropoiesis in the bone marrow, with a later decline in primitive hematopoietic cells.(34,35) Additionally, cells of the osteoblast lineage seem to play an active role in HSC mobilization, for instance in response to granulocyte-colony stimulating factor (G-CSF),(36,37) although their precise role is still being determined.

Genetic manipulation of either the PTH/PTH-related peptide receptor (PPR) in osteoblasts(38) or the BMPR1a receptor(39) leads to increased osteoblast number and enhanced HSC frequency. However, osteoblast number is not the sole determinant of the number of HSCs, because the reduced osteoblast number seen in biglycan knockout (KO) mice is not associated with a decrease in HSCs or any other hematopoietic defect.(40) Several factors elaborated or influenced by osteoblasts have been identified that can regulate hematopoiesis, including angiopoietin-1,(41) osteopontin,(42,43) thrombopoietin,(44) Wnts,(45) and extracellular calcium.(46) Notch signaling has been implicated by the finding that increased expression of the Notch ligand Jagged-1 (Jag1) in osteoblasts after PPR activation is associated with an increase in HSC number and that this increase in HSCs could be blocked by administration of a γ-secretase inhibitor,(38) although Mx1-Cre-mediated deletion of Jag1 in the microenvironment has been reported to yield no phenotype.(47) Some studies have suggested the importance of N-cadherin,(8,39),(48) whereas others have disputed the relevance of N-cadherin.(40) Thus, whereas osteoblasts clearly have a role in the establishment of an HSC niche, the molecular mechanisms remain incompletely defined, and there are many unanswered questions. In particular, a requirement for direct contact between HSCs and osteoblasts, as opposed to proximity to diffusible factors, has yet to be definitively shown in vivo, and the precise characterization of the osteoblasts capable of supporting HSCs is very incomplete. Furthermore, the interplay between cells of the osteoblast lineage and perivascular/vascular cells in the bone marrow niche are largely unknown. In the future, cell-specific ablation of candidate factors will be useful in clarifying many of these issues.

## DISTINCT NICHES EXIST WITHIN THE BONE MARROW FOR DEVELOPING B CELLS

Within the bone marrow, specific niches have also been identified for maturing hematopoietic cells, including platelets(49) and B lymphocytes. B-lymphocyte development is well characterized, and several studies now point to the existence of distinct niches for each stage of differentiation. B lymphocytes are generated from HSCs through a common lymphoid progenitor (CLP), and within the bone marrow, the earliest identifiable B-cell precursor is the prepro-B cell. Prepro-B cells differentiate into pro-B cells, which in turn gives rise to pre-B cells.(50) Immature IgM^+^ B cells migrate into the periphery, where maturation occurs in the spleen. Tokoyoda et al.(51) found that, within bone marrow stroma, CXCL12 and interleukin (IL)-7, two factors with critical roles at differing stages of B lymphopoiesis, are expressed by separate stromal cell populations. Whereas prepro-B cells are in contact with CXCL12-expressing stromal cells, more differentiated pro-B cells instead are found in contact with IL-7–expressing cells, suggesting that as B-cell precursors differentiate, they migrate among discrete populations of stromal cells. Finally, pre-B cells do not contact either CXCL12- or IL-7–expressing cells, consistent with the finding that in vitro pre-B cells are no longer dependent on stromal cell support. Intriguingly, end-stage plasma cells return to the bone marrow, where dendritic cells provide a supportive niche,(52) further supporting the possibility that each stage of B-lymphocyte differentiation may rely on a specific niche.

## G_S_α MEDIATES OSTEOBLASTIC REGULATION OF B-CELL DEVELOPMENT

In addition to hematopoietic progenitors, the bone marrow contains stromal cells that include precursors of the osteoblast lineage. Both CXCL12 and IL-7 can be produced by cells of the osteoblast lineage, and both are upregulated in response to PPR-mediated signaling.(35,38),(53) This raises the possibility that either or both populations of stromal cells expressing CXCL12 or IL-7 may share characteristics with cells of the osteoblast lineage. Consistent with this model, Zhu et al.(35) found that primary calvarial cells, which include osteoblasts and their progenitors, are capable of supporting B lymphopoiesis in culture, and that this supportive ability is enhanced by treatment with PTH.

The PPR is a G protein-coupled receptor (GPCR), and the heterotrimeric G protein subunit G_s_α is a major downstream mediator of PPR signaling through the protein kinase A pathway.(54) We have deleted G_s_α in early osteoprogenitors using Cre recombinase driven by osterix, a transcription factor expressed early in osteoblastogenesis. G_s_α^OsxKO^ mice have a dramatic reduction in trabecular bone.(55) Because enhanced signaling through PPR is associated with increased HSC number,(38) and because PTH can also stimulate osteoblastic support of B-lymphocyte differentiation,(35) we asked whether loss of G_s_α would conversely have a negative impact on hematopoiesis. In fact, G_s_α^OsxKO^ mice have a significant decrease in B-cell precursors in the bone marrow, and this results in a reduction in circulating B lymphocytes.(55) Within the bone marrow, B lymphopoiesis is impaired at the pro-B to pre-B cell transition, whereas earlier prepro-B cells are unaffected. Consistent with the finding that prepro-B cells are associated with CXCL12^+^ cells, CXCL12 expression is not reduced in G_s_α^OsxKO^ osteoblasts. In contrast, IL-7 mRNA is significantly decreased, and the reduction in pro-B and pre-B cells is similar to that found in mice lacking either IL-7 or the IL-7 receptor.(56,57) Moreover, exogenous IL-7 is sufficient to rescue the pro-B deficit in G_s_α^OsxKO^ mice. The B-lymphocyte deficiency can be rescued by transplanting KO bone marrow into a wildtype host, confirming that this is caused by a defect in the microenvironment. Thus, G_s_α signaling within cells of the osteoblast lineage is required for normal bone marrow B lymphopoiesis and likely involves IL-7 production. Candidate G_s_α-coupled GPCRs in the osteoblast lineage that might regulate B-lymphocyte development include PPR and the prostaglandin E_2_ receptors EP2R and EP4R.(58) Of note, prostaglandin E_2_ has also been reported to modulate the HSC niche.(59)

## MODEL FOR OSTEOBLASTIC SUPPORT OF HEMATOPOIETIC DEVELOPMENT

To summarize, within the bone marrow, cells of the osteoblast lineage have unequivocally been shown to constitute a niche for HSCs and now have been found to support differentiation of the B-lymphocyte lineage as well. Ablation of G_s_α early in the osteoblast lineage results in loss of pro-B cells and is associated with decreased expression of IL-7 in cells expressing osterix.(55) Because IL-7^+^ cells are found in the marrow and are distinct from mature endosteal osteoblasts,(51) perhaps IL-7–expressing stromal cells represent a subset of cells of the osteoblast lineage involved in supporting hematopoiesis.

**FIG. 1:**
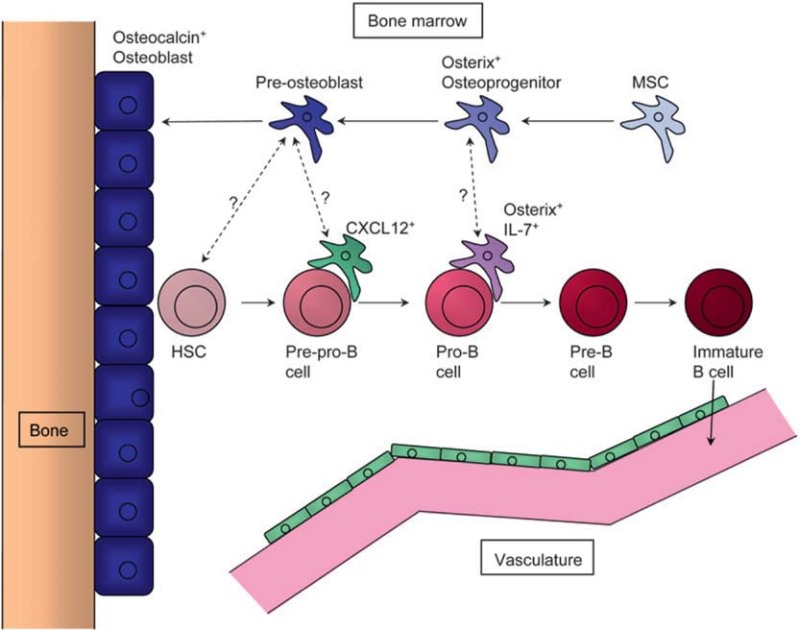
Model for the role of the osteoblast lineage in the bone marrow hematopoietic niches. Cells of the osteoblast lineage are in blue, whereas hematopoietic cells are red. Within each lineage, darker color intensity reflects a more advanced stage of differentiation. In this model, mature osteocalcin-expressing osteoblasts provide a niche for HSCs, whereas more-differentiated pro-B cells are supported by cells expressing both osterix and IL-7. However, many questions await further study. Are the osterix^+^IL-7^+^ cells the same as osterix^+^ osteoprogenitors? Cells of the osteoblast lineage can produce CXCL12; are CXCL12^+^ cells that contact prepro-B cells a subset of the osteoblast lineage? Finally, do HSCs require direct contact with mature osteoblasts and do earlier cells in the osteoblast lineage have any role in supporting HSCs?

Based on the evidence to date regarding the role of osteoblast lineage cells in the bone marrow niche, we propose that cells derived from osteoprogenitors and in various stages of differentiation provide distinct niches for hematopoietic cells. Thus, as others have postulated,(3–5,60) terminally differentiated osteoblasts along the endosteal surface would serve as a niche for HSCs, perhaps in their most quiescent stage.(61) Once committed to the B-cell lineage, prepro-B cells migrate to CXCL12^+^ cells. Differentiation into pro-B cells requires IL-7, produced by osteoblast lineage cells expressing osterix. Thus, the earliest hematopoietic progenitor, the HSC, is supported by mature osteoblasts, whereas more differentiated pro-B cells are themselves supported by osteoprogenitors located in the marrow space (Fig. 1). In support of the concept that mature osteoblasts and stromal osteoprogenitors have differential effects on hematopoietic cells, Balduino et al.(62) found that both osteoblasts and subendosteal reticular cells express osteogenic markers. However, whereas osteoblasts maintain hematopoietic progenitors with low proliferation, the stromal reticular cell fraction, which likely includes osteoprogenitors, induces proliferation and differentiation of hematopoietic cells. One prediction of this model is that CXCL12(hi)-reticular cells,(60) which contact intermediary prepro-B cells, might also represent a subset of the osteoblast lineage. IL-7^+^Osx^+^ cells and CXCL12(hi) cells may or may not be the same population as those Osx^+^ cells that ultimately give rise to terminally differentiated osteoblasts. Much work remains to be done to confirm such a model, but it provides a framework with which to begin to dissect the mechanisms of cross-talk between the skeletal and hematopoietic systems. In addition, whether cells of the osteoblast lineage play any role in supporting hematopoietic lineages other than B lymphocytes is still unknown.

## CLINICAL RELEVANCE

The role of the microenvironment in disease pathogenesis is garnering increasing attention, with several recent reports highlighting the importance of the microenvironment on neoplasia.(63–66) It has long been known clinically that many malignancies display a predilection for metastasis to bone, and whether these predilections involve the same molecular mechanisms as niche interactions is an area of intense interest. With respect to B-cell lineage malignancies, multiple myeloma bone disease is a devastating complication that involves interactions between malignant plasma cells, osteoblasts, and osteoclasts.(67) In a model of human MLL-AF9 leukemia, myeloid versus lymphoid lineage could be directed by changes in the microenvironment.(68) How myeloid malignancies are influenced by osteoblasts is at present unknown. However, human leukemic cells transplanted into NOD/SCID mice home to osteoblasts,(69,70) strongly supporting a functional relationship and suggesting that intervening in such an interaction may be a worthy area of study.

## CONCLUDING REMARKS

Because of the very nature of the bone marrow niche, at the intersection of hematopoietic, skeletal, and vascular biology, approaches to this field have been varied, reflecting the diverse origins of the investigators. As we move forward, there will be a need to reconcile anatomic, genetic, and functional data to better delineate the relative contributions and roles of the myriad components. For example, HSCs have been variably identified by immunophenotype, immunohistochemical localization, and functional studies. HSCs themselves may also exert some influence on the microenvironment, because HSCs have been shown to exert stimulatory effects on osteogenic differentiation of bone marrow stromal cells in a co-culture system.(71) In addition, bone-resorbing osteoclasts, derived from the macrophage/monocyte lineage,(72) have been implicated in the regulation of the HSC niche,(73) highlighting the complexity of cross-talk between an ever-growing list of participants. On the stromal side, a variety of promoters have been used to dissect the microenvironment, and a better understanding of the spatial and temporal relationships between markers expressed by these cell populations is needed. As an example, Mx1-Cre is commonly used in combination with reciprocal transplantation studies to elucidate the relative intrinsic versus extrinsic contributions of various gene mutations to hematopoietic phenotypes. However, the expression of Mx1-Cre within the stromal microenvironment and its relative efficacy in different stromal cell compartments remains obscure. In particular, although Mx1 is expressed in the skeleton,(74) phenotypes resulting from Mx1-Cre-mediated deletion differ from those obtained with osteoblast-specific promoter-driven Cre recombinases. Deletion of Rb in the microenvironment with Mx1 leads to myeloproliferative disease,(63) whereas deletion of Rb within the osteoblast lineage results in enhanced predilection for development of osteosarcoma.(75)

In summary, osteoblasts cannot be considered as a uniform entity, but rather as a complex population of cells with a broad spectrum of developmental potential. A better understanding of how anatomic localization, immunophenotype, and stage of differentiation and cellular function are interrelated will be crucial to advance the biology of osteoblasts within the bone marrow niche.
